# Imaging of Carotid Blowout Syndrome in a Patient with Nasopharyngeal Carcinoma after Radiation Therapy

**DOI:** 10.2174/0115734056407874250916054722

**Published:** 2025-10-06

**Authors:** Yuanling Yang, Xinting Peng, Weiyi Liu, Lixuan Huang, Zisan Zeng

**Affiliations:** 1 Department of Radiology, The First Affiliated Hospital of Guangxi Medical University, Nanning, 530021, Guangxi, China

**Keywords:** Carotid blowout syndrome, MRA, Carotid artery, Nasopharyngeal carcinoma, MRI, Vascular complications, Computed tomography angiography

## Abstract

**Introduction:**

This case highlights the rare but life-threatening complication of carotid blowout syndrome (CBS) after radiotherapy for nasopharyngeal carcinoma (NPC). It is characterized by rupture of the carotid artery, often occurring months or years after treatment. Early diagnosis and timely intervention are essential to improve clinical outcomes.

**Case Presentation:**

A 45-year-old woman with NPC developed recurrent epistaxis 31 months after chemoradiotherapy. MRI and MRA ruled out tumor recurrence. High-resolution vessel wall imaging (VWI) revealed eccentric thickening, irregular enhancement, and a pseudoaneurysm in the lacerum segment of the left internal carotid artery (ICA), which was confirmed by CTA and DSA. The patient underwent embolization and remained stable at 1-year follow-up.

**Conclusion:**

This case underscores the value of VWI in detecting CBS-related vascular changes. Imaging is crucial for early diagnosis and timely intervention in high-risk patients with NPC who have undergone radiotherapy.

## 1 INTRODUCTION

Nasopharyngeal carcinoma (NPC) is highly prevalent in South China, Southeast Asia, and North Africa. Radiation therapy is the preferred and only curative method for NPC [1, 2]. However, as the cure rate rises, the adverse effects of radiation have become a major concern. Carotid blowout syndrome (CBS) is defined as rupture of the carotid artery and its main branches. This condition is a rare but devastating complication of radiotherapy for NPC [3, 4]. Therefore, recognizing and analyzing the imaging features of patients with CBS is crucial for identifying individuals who may be at high risk of future bleeding. We present a case detailing the imaging features of a patient with NPC who developed CBS after radiotherapy, particularly high-resolution vessel wall imaging (VWI) features, offering a more comprehensive understanding of CBS diagnosis. Importantly, this case also highlights the complementary role of VWI with angiography for evaluating patients with radiation-associated vascular complications.

## 2 CASE PRESENTATION

Our patient was a 45-year-old female without hypertension, hyperlipidemia, diabetes, and overweight, and was diagnosed with NPC in July 2021 (stage T4N2M0, IVa stage using the 8^th^ edition UICC/AJCC TNM staging system). The patient received induction chemotherapy and concurrent chemoradiotherapy according to the guidelines of the Radiation Therapy Oncology Group. The radiotherapy dose was PTVnx: 72Gy; PTVnd: 68Gy; PTV1: 62Gy; PTV2: 54Gy.

She was readmitted to the hospital with epistaxis 31 months after completing chemoradiotherapy for NPC. The physical examination revealed vital signs: body temperature, 36.1°C; pulse rate, 92/min; respiration rate, 20/min; and blood pressure, 110/80 mmHg. The patient was conscious with stable breathing. Superficial lymph nodes were not enlarged; lungs were clear, and the heart rhythm was regular. The abdomen was flat, soft, with no tenderness, and no lower limb edema. At our institution, MRI and MRA are routinely used as first-line imaging modalities for surveillance of tumor recurrence and vascular status in NPC patients following radiotherapy. In this case, conventional MRI showed no evidence of tumor recurrence, and MRA demonstrated no significant vascular abnormalities. However, given the persistence of unexplained epistaxis and clinical concern for vascular pathology, further evaluation with high-resolution vessel wall imaging (VWI) and computed tomography angiography (CTA) was performed.

VWI was performed on a 3.0T MRI scanner (Magnetom Prisma-fit, Siemens Healthcare, Erlangen, Germany) using the following sequences: a conventional axial T2-weighted turbo spin-echo sequence (T2-TSE_tra; TR/TE = 7510/77 ms, slice thickness = 5.0 mm, FOV = 220 mm) and a Dixon-based fast T2-weighted TSE sequence (T2_TSE_Dixon_tra_p2_320_fast; TR/TE = 4310/81 ms, slice thickness = 5.0 mm, FOV = 245 mm). Intravenous gadoteric acid meglumine (0.2 mL/kg body weight) was administered for contrast-enhanced imaging. Imaging revealed eccentric wall thickening and heterogeneous contrast enhancement involving the perivascular space and the lacerum segment of the bilateral internal carotid arteries (ICA), indicative of inflammatory vasculopathy.

**Fig. (1):**
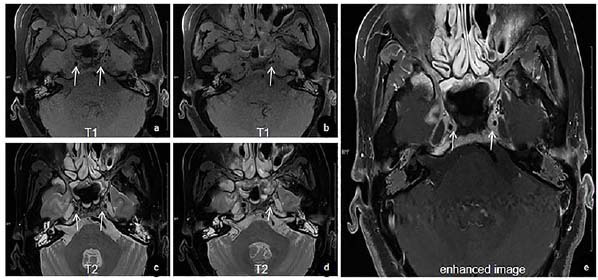
High-resolution vessel wall imaging shows concentric thickening in the right ICA and eccentric thickening in the left ICA **(**a**, **c**, **e**)**, double lumen in the left ICA **(**b**, **d**)**. ICA: internal carotid artery.

**Fig. (2):**
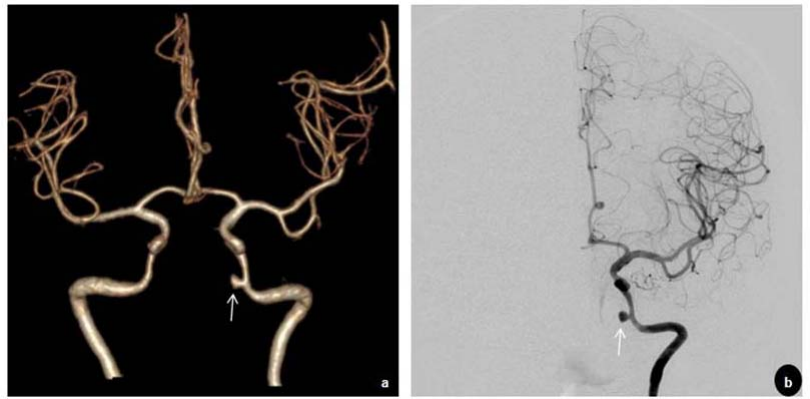
A capsule-like protrusion (arrow) at the internal carotid artery is identified on the computed tomography angiography **(a)** and digital subtraction angiography images **(b)**.

Additionally, pseudoaneurysm formation was observed in the left ICA (Fig. **1**). Computed tomography angiography (CTA) revealed an aneurysm formation in the ruptured lacerum segment of the left ICA (Fig. **2a**). Considering that digital subtraction angiography (DSA) is the gold standard for the clinical diagnosis of CBS, the patient underwent DSA, which identified a saccular aneurysm in the lacerum segment of the left ICA (Fig. **2b**), measuring approximately 4.5 mm × 3.1 mm with a neck diameter of 3.6 mm. The balloon occlusion test demonstrated adequate collateral flow through the anterior communicating artery but insufficient perfusion in the posterior circulation. Aneurysm embolization was successfully performed without complications, such as significant bleeding or subcutaneous hematoma at the puncture site. At the 1-year postoperative follow-up, the patient showed no associated complications or signs of tumor recurrence.

Radiation-induced vascular injury can cause arterial stenosis or occlusion, vasculitis, thrombosis, and aneurysm formation. Pseudoaneurysms are commonly observed in various types of CBS. The patient’s culprit artery is located in the radiation field and received the radiation dose, supporting the diagnosis of radiation-induced vasculopathy.

## 3 DISCUSSION

This article reports on the imaging findings of radiation-induced CBS in a patient with NPC. Reports indicate that ICA rupture is the primary cause of massive hemorrhage in nasopharyngeal bleeding following NPC treatment [5]. During the treatment of NPC, local inflammation or residual tumor tissue often causes different degrees of tissue necrosis and infection. Tissue fibrosis following radiotherapy (RT) hinders effective tissue repair, and concurrent infections exacerbate the necrosis and ulceration, ultimately exposing the arterial vessels. When NPC lesions erode the ICA, tissue necrosis and ulceration after RT can cause great damage to its vessel wall [6]. Due to long-term inflammation and infectious infiltration, the blood vessel wall is continuously damaged, which may result in serious bleeding events. RT induces the degeneration and necrosis of osteocytes, leading to skull base osteonecrosis. Meanwhile, the increased oxygen demand caused by infection further exacerbates bone necrosis. Extensive necrosis of the skull base exposes the major nasopharyngeal vessels, compromising their support and heightening the risk of CBS [7].

CBS is classified into three subtypes: Type I CBS is characterized by carotid artery exposure during examination or imaging; Type II CBS is marked by bleeding episodes or sentinel bleeding; and Type III CBS is defined by fatal hemorrhage in the carotid system [8]. Research reports that the incidence of massive epistaxis ranges from 5 to 11% [9-11]. Although its incidence is low, massive hemorrhage resulting from CBS is acute and life-threatening, with a high mortality rate if not treated promptly. The morbidity and mortality rates associated with CBS are reported to be approximately 40% and 60%, respectively, and the survival rate is generally < 2 years [12], underscoring the need for heightened vigilance.

The identification of the bleeding site typically relies on clinical presentation and prior MRI or CT imaging of the affected region [13]. MRI plays a pivotal role in detecting and monitoring vascular lesions of both intracranial and extracranial origins. It not only provides a detailed visualization of the vascular lumen but also enables precise evaluation of the vessel wall, aiding in the differentiation of various vascular pathologies. Guggenberger *et al*. underscored the effectiveness of high-resolution, compressed-sensing, black-blood 3D T1-weighted fast spin-echo imaging for assessing a wide spectrum of vascular conditions in radiology [14]. CTA can noninvasively assess the condition of pathological vessels in patients with episodic but clinically stable CBS, without increasing the risk of stroke, and provides rapid prognostic insights. This can preliminarily distinguish between soft tissue source and vascular hemorrhage [15]. DSA offers superior accuracy compared to other modalities in identifying the site of CBS and serves as the gold standard for its clinical diagnosis. Additionally, DSA provides real-time assessment of treatment efficacy [16]. Yang *et al*. identified several important risk factors for CBS bleeding, including ICA injury, artery exposure, sphenoid sinusitis, and skull base osteonecrosis, with the latter two being particularly significant. Appropriate examination or treatment should be implemented in such cases [17]. In our case, T2-weighted VWI revealed sphenoid sinus effusion, indicating sphenoid sinusitis. Vascular stenosis was observed in T2- and T1-weighted unenhanced VWI images on both sides of the ICA, while thickening and enhancement of the vascular wall indicated vascular inflammation. Additionally, pseudoaneurysm formation was noted in the left ICA. These findings highlight the diagnostic value of VWI in detecting early vessel wall changes and radiation-induced vasculopathy. Given its ability to noninvasively characterize vascular inflammation and mural abnormalities, VWI holds promise as a valuable tool for routine surveillance in high-risk NPC patients following radiotherapy. Furthermore, CT revealed skull base osteonecrosis, and CTA indicated aneurysm formation in the ruptured lacerum segment of the left ICA, similar to the results of a previous study [17].

Direct imaging signs of CBS on DSA may include irregular arterial walls, lumen stenosis, pseudoaneurysm, contrast extravasation, and arterial wall rupture [18]. In our case, the most pronounced damage involved the intracranial segments of the bilateral ICAs, with clear evidence of CBS on the left side, manifested by a radiation-induced pseudoaneurysm. A double lumen sign was also noted in the affected segment, which is considered a characteristic indicator of vessel wall dissection or intramural hematoma, and may represent an early stage of pseudoaneurysm formation. Recognizing such subtle imaging findings is critical for the timely diagnosis and management of radiation-related vascular complications. A previous study demonstrated that the presence of air-containing necrosis is a major risk factor for CBS [19]. However, this radiological feature was not observed in the present case. This suggests that post-radiotherapy neck vascular lesions may exhibit a spectrum of diverse imaging features, necessitating a comprehensive analysis of various imaging modalities for accurate diagnosis.

Endovascular therapy is an ideal approach for managing CBS resulting from the ICA. It can also prevent acute carotid ruptures in patients undergoing reirradiation for recurrent head and neck cancers and effectively treat recurrent epistaxis in NPC patients [20]. DSA provides clear visualization of abnormal blood vessels, allowing for the simultaneous treatment of damaged areas, and is therefore the preferred method for addressing pseudoaneurysms after NPC radiotherapy [1]. Additionally, DSA-guided selective arterial embolization offers significant hemostatic effects, making it a safe and effective option for the clinical diagnosis and management of major bleeding following NPC radiotherapy, as well as the first-line treatment for controlling such bleeding [21]. Prior to the procedure, thorough discussions were held with the patient and their family regarding the available treatment options and potential risks. After obtaining informed consent, the patient and family opted for interventional treatment, and aneurysm embolization was performed. Postoperative management included antiplatelet therapy, fluid resuscitation, nebulization therapy, and symptomatic support. At the 1-year follow-up, the patient demonstrated no complications or signs of tumor recurrence.

MRI and CTA can noninvasively assess the status of diseased vessels in patients with episodic but clinically stable CBS, without increasing the risk of stroke, and can rapidly provide prognostic information. Patients who have undergone neck irradiation require meticulous scrutiny and thorough diagnosis of their cervical vascular lesions, along with heightened awareness of this potential complication. Furthermore, consistent long-term surveillance and monitoring of disease progression post-radiotherapy are imperative. Prompt imaging assessments, such as MRI or CTA, should be performed for patients exhibiting clinical symptoms to ensure timely detection and diagnosis of potential vascular lesions, thereby reducing the morbidity and mortality associated with CBS.

This case report has several limitations. Being a single case, its findings may not be representative of the broader population of NPC patients with post-radiotherapy CBS. Although the patient underwent 12 months of follow-up, surveillance relied on conventional MRI without serial VWI, which restricted the evaluation of vascular lesion progression. The absence of quantitative vessel wall analysis limits the reproducibility and objective assessment of imaging features. Furthermore, without comparison to non-radiation-induced vascular pathology, the specificity of these findings remains uncertain.

## CONCLUSION

Imaging evaluations play a critical role in the early diagnosis and management of CBS in patients with NPC following radiation therapy. It is essential to maintain vigilance regarding changes in carotid artery imaging post-radiotherapy to effectively mitigate the risk of complications.

## AUTHORS’ CONTRIBUTIONS

The authors confirm their contributions to the paper as follows: Y.Y. and Z.Z.: Study concepts and design were developed; X.P.: Data collection was conducted; W.L.: Analysis and interpretation of results were performed; Y.Y. and L.H.: Manuscript was drafted. All authors reviewed the results and approved the final version of the manuscript.

## Data Availability

All data generated or analyzed during this study are included in this published article.

## LIST OF ABBREVIATIONS

NPC: Nasopharyngeal carcinoma
CBS: Carotid blowout syndrome
ICA: Internal carotid artery
VWI: Vessel wall imaging
CTA: Computed tomography angiography
DSA: Digital subtraction angiography
MRI: Magnetic resonance imaging
MRA: Magnetic resonance angiography
RT: Radiotherapy
PTVnx: Planning Target Volume of the primary nasopharyngeal tumor
PTVnd: Planning Target Volume of the involved neck nodes
PTV1: Planning Target Volume of high-risk clinical target volume
PTV2: Planning Target Volume of low-risk clinical target volume
AJCC: American Joint Committee on Cancer
UICC: Union for International Cancer Control

## STANDARDS OF REPORTING

CARE guidelines were followed.
